# The association of CD40 polymorphisms with CD40 serum levels and risk of systemic lupus erythematosus

**DOI:** 10.1186/s12863-015-0279-8

**Published:** 2015-10-16

**Authors:** Jian-Ming Chen, Jing Guo, Chuan-Dong Wei, Chun-Fang Wang, Hong-Cheng Luo, Ye-Sheng Wei, Yan Lan

**Affiliations:** Department of Laboratory Medicine, Affiliated hospital of Youjiang Medical University for Nationalities, Baise, 533000 Guangxi China; Department of Dermatology, Affiliated Hospital of Youjiang Medical University for Nationalities, Baise, 533000 Guangxi China

**Keywords:** CD40, Gene, Polymorphism, SLE

## Abstract

**Background:**

Current evidence shows that the CD40–CD40 ligand (CD40–CD40L) system plays a crucial role in the development, progression and outcome of systemic lupus erythematosus (SLE). The aim of this study was to investigate whether a CD40 gene single nucleotide polymorphism (SNP) is associated with SLE and CD40 expression in the Chinese population. We included controls (*n* = 220) and patients with either SLE (*n* =205) in the study.

**Methods:**

The gene polymorphism was measured using Snapshot SNP genotyping assays and confirmed by sequencing. We analyzed three single nucleotide polymorphisms of CD40 gene rs1883832C/T, rs1569723A/C and rs4810485G/T in 205 patients with SLE and 220 age-and sex-matched controls. Soluble CD40 (sCD40) levels were measured by ELISA.

**Results:**

There were significant differences in the genotype and allele frequencies of CD40 gene rs1883832C/T polymorphism between the group of patients with SLE and the control group (*P* < 0.05). sCD40 levels were increased in patients with SLE compared with controls (*P* < 0.01). Moreover, genotypes carrying the CD40 rs1883832 C/T variant allele were associated with increased CD40 levels compared to the homozygous wild-type genotype in patients with SLE. The rs1883832C/T polymorphism of CD40 and its sCD40 levels were associated with SLE in the Chinese population.

**Conclusions:**

Our results suggest that CD40 gene may play a role in the development of SLE in the Chinese population.

## Background

Systemic lupus erythematosus (SLE) a kind of chronic autoimmune disease, leading to multiple organ damage, has the characteristics of various autoantibodies production. Although that the etiology and pathogenesis of SLE is not clear, it maybe immune regulation disorder caused by a complex interplay of genetic and environmental factors, hormones, antigen antibody and complement complex deposits lead to local or systemic tissue or organ damage [1–4]. Among them, genetic factors seem to play a key role in the susceptibility to SLE. In the past several years genome-wide association studies (GWAS) for SLE have identified literally hundreds of genetic loci involved in the susceptibility conferred to complex inherited traits [5–7]. Even though this scenario represents an extraordinary advance in complex disease genetics, the modest effect sizes of the common polymorphisms found associated explain only a small fraction of the heritability in most of these multifactorial conditions, suggesting that many more loci remain to be discovered [8, 9]. One of the genes encoding a member of the tumor necrosis factor receptor family that plays a key role in adaptive immunity of SLE is CD40 [10].

CD40, a member of the tumor necrosis family of transmembrane glycoproteins, was identified on B cells, monocytes, dendritic cells, endothelial and epithelial cells, which is rapidly and transiently expressed on the surface of recently activated CD4^+^T cells and is a potent T-cell costimulatory molecule [11–13]. Interactions between CD40 and CD40L induce B cell immunoglobulin production as well as monocyte activation and dendrite cell differentiation [14, 15]. Some authors have demonstrated that the multipotent immunomodulator CD40, expressed on vascular endothelial cells, smooth muscle cells, mononuclear phagocytes, and platelets, promote awide array of pro-atherogenic functions in vitro [16–19]. The gene encoding CD40 is located on chromosome 20q11-13 in humans, which consists of nine exons and eight Introns. Recently, a number of polymorphisms in the gene encoding CD40 gene have been identified and a relationship between the CD40 gene polymorphisms and risk of different autoimmune and inflammatory diseases, such as multiple autoimmune diseases, Graves’ disease and rheumatoid arthritis has been reported [20–22]. However, very little data has examined the association between rs1883832C/T, rs1569723A/C and rs4810485G/T polymorphisms in CD40 gene and SLE. Furthermore, the relationship between the CD40 gene polymorphisms and the plasma level of CD40 gene is unknown. In this study, we investigated the relationship of CD40 gene rs1883832C/T, rs1569723A/C and rs4810485G/T polymorphisms and their CD40 serum levels in a Chinese population.

## Methods

### Study population

Our study was designed as a retrospective study. The study consisted of 205 patients with SLE (36 males and 169 females, aged between 30 and 82 years). All patients with SLE were consecutively selected. They were recruited from the Department of Dermatology, Affiliated Hospital of Youjiang Medical University for Nationalities, Guangxi, China between October 2014 and November 2015. The 220 control subjects were matched to the patients on the basis of age and gender (42 males and 178 females, aged between 29 and 78 years). The control subjects underwent a routine medical check-up in the outpatient clinic of the Department of Internal Medicine, Affiliated Hospital of Youjiang Medical University for Nationalities, Guangxi, China between May 2013 and November 2014. According to the thorough clinical and laboratory evaluation, none of them were found to have any medical condition other than hypertension, autoimmune and inflammatory diseases. All study subjects were Chinese and resided in the same geographic area in China. The study was performed with the approval of the ethics committee of the Affiliated Hospital of Youjiang Medical University for Nationalities, and written informed consent was obtained from all the subjects.

### DNA extraction

Genomic DNA was extracted from EDTA-anticoagulated peripheral blood leukocytes by the salting-out method [23]. Briefly, 3 ml of blood was mixed with Triton lysis buffer (0.32 M sucrose, 1 % Triton X100, 5 mM MgCl_2_, H_2_O, 10 mM Tris–HCl, pH 7.5). Leucocytes were spun down and washed with H_2_O. The pellet was incubated with proteinase K at 56 °C and subsequently salted out at 4 °C using a saturated NaCl solution. Precipitated proteins were removed by centrifugation. The DNA in the supernatant fluid was dissolved in 300 μlH_2_O.

### Determination of CD40 genotype

The CD40 gene rs1883832 C/T, rs1569723 A/C and rs4810485 G/T genotypes were determined by using a Snapshot SNP genotyping assay. The PCR primers were designed based on the GenBank reference sequence (accession no. NC_000020.11) (Table 1). To confirm the genotyping results, PCR-amplified DNA samples were examined by DNA sequencing, and the results were 100 % concordant.

**Table 1:** The primer sequences used for detecting the different CD40 SNPs

Reference SNP ID	PCR primers
rs1883832C/T	F:5'-GGACCTGGGGGCAAAGAAGA-3'
	R: 5'- CCCACTCCCAACTCCCGTCT -3'
	EF:5'-TTTTTTTTTTTTGCAGAGGCAGAC
	GAACCAT -3'
rs1569723 A/C	F: 5'- GGGATG GCCTGATCCAAAGG -3'
	R: 5'- CCCACAGTCCACCACCCATC -3'
	EF:5'-TTTTTTTTTTTTTTTTTTTTTTTTTTTT
	TTTTTCGCTTTACACCCACAGCC-3'
rs4810485 G/T	F: 5'- ATCCCCCAAGTACCTGGCTCCT -3'
	R: 5'- CCTTGCTGCTTCCC TTGCTTTC -3'
	EF:5'- TTTTTTTTTTTTTTTTTTTTTTTTTTT
	TCCTACTTTAGAG GGCTGTAGATTCC -3'

### Plasma CD40 determination

Plasma samples from the patients and healthy controls were separated from venous blood at room temperature, and stored at −70 °C until use. The quantity determination of plasma CD40 levels was performed by enzyme-linked immunosorbent assay (ELISA) kits (Fermentas, Lithuania), following the manufacturer’s protocol. Developed color reaction was measured as OD450 units on an ELISA reader (RT-6000, China). The concentration of plasma CD40 was determined by using standard curve constructed with the kit’s standards over the range of 0–1000 pg/ml.

### Statistical analysis

Genotype and allele frequencies of CD40 were compared between SLE cases and controls using the *χ*2 test and Fisher’s exact test when appropriate, and odds ratios (OR) and 95 % confidence intervals (CIs) were calculated to assess the relative risk conferred by a particular allele and genotype. Demographic and clinical data between groups were compared by *χ*2 test and by Student’s *t*-test. Hardy–Weinberg equilibrium was tested for with a goodness of fit *χ*2-test with 1 ° of freedom to compare the observed genotype frequencies among the subjects with the expected genotype frequencies. The linkage disequilibrium (LD) between the polymorphisms was quantified using the Shi’s standardized coefficient D' (|D'|) [24]. The haplotypes and their frequencies were estimated based on a Bayesian algorithm using the Phase program [25]. Statistical significance was assumed at the *P* < 0.05 level. The SPSS statistical software package version 11.5 was used for all of the statistical analysis.

## Results

### Clinical characteristics of the study participants

**Fig. 1:**
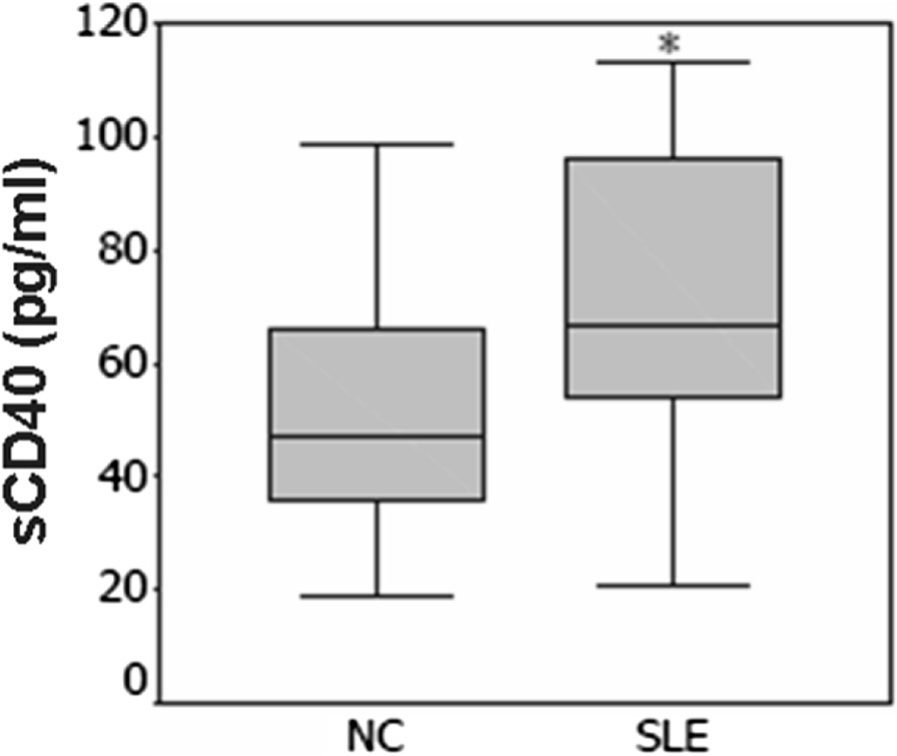
The levels of CD40 in patients with SLE and normal control subjects. The expression of CD40 was significantly increased in patients with SLE compared to that in control subjects [(mean +/− SD 41.7+/− 13.2 pg/ml, *n* = 205) vs (mean +/− SD 58.5+/− 22.8 pg/ml, *n* = 220); *P* <0.001]

There were no significant differences in the age (*P* > 0.05) and percentage of males/females (*P* > 0.05) between the two groups. The serum CD40 levels were significantly higher in the group of patients with SLE than those in the control group [(mean +/− SD 58.5 +/− 22.8 pg/ml, *n* = 205) vs, (mean +/− SD 41.7 +/− 13.2 pg/ml, *n* = 220); *P* <0.001] (Fig. 1).

### The genotype and allele frequencies of CD40 gene

The genotype and allele frequencies of the CD40 gene rs1883832 C/T, rs1569723 A/C and rs4810485 G/T polymorphisms in the group of patients with SLE and in the control group are shown in Table 2. The genotype distributions of the three polymorphisms among the controls and the cases were in Hardy–Weinberg equilibrium, and the Hardy–Weinberg equilibrium p-values of the CC, CT and TT genotypes of rs1883832 C/T, rs1569723 A/C and rs4810485 G/T were 0.440, 0.509 and 0.686 in controls, and were 0.718, 0.195 and 0.300 in cases, respectively. The frequencies of the CC, CT and TT genotypes of rs1883832 C/T were 35.9, 45.9 and 18.2 % in controls, and were 22.9, 51.2 and 25.9 % in cases, respectively. There were significant differences in the genotype and allele frequencies of the CD40 gene rs1883832 C/T polymorphism between the SLE and control groups. The rs1883832 T allele was associated with a significantly increased risk of SLE as compared with the rs1883832 C allele (OR = 1.517, 95 % CI, 1.157–1.990, *P* = 0.003). However, genotype and allele frequencies of the CD40 gene rs1569723 A/C and rs4810485 G/T polymorphisms in SLE patients were not significantly different than those in controls (*P* > 0.05).

**Table 2:** The genotype and allele frequencies of CD40 polymorphism in SLE patients and controls

Polymorphism	Control subjects *n* = 220 (%)	SLE patients *n* = 205 (%)	*χ* ^*2*^	*P* value
rs1883832 C/T				
CC	79 (35.9)	47 (22.9)	9.504	0.009
CT	101 (45.9)	105 (51.2)		
TT	40 (18.2)	53 (25.9)		
C	259 (58.9)	199 (48.5)	9.109	0.003
T	181 (41.1)	211 (51.5)		
rs1569723 A/C				
AA	54 (24.5)	51 (24.9)	0.284	0.868
AC	105 (47.7)	93 (45.4)		
CC	61 (27.7)	61 (29.8)		
A	213 (48.4)	195 (47.6)	0.061	0.805
C	227 (51.6)	215 (52.4)		
rs4810485 G/T				
GG	56 (25.5)	52 (25.4)	0.341	0.843
GT	107 (48.6)	95 (46.3)		
TT	57 (25.9)	58 (28.3)		
G	219 (49.8)	199 (48.5)	0.130	0.719
T	221 (50.2)	211 (51.5)		

### Haplotype analysis of the CD40 gene

Haplotype analyses were performed and the possible six haplotype frequencies are shown in Table 3. Two major haplotypes (TCT and CAG) accounted for 51.5, 42.9 and 43.0, 47.0 % of these six haplotypes in both the cases and the controls, respectively. CD40 gene rs1883832 C/T polymorphism was in strong linkage disequilibrium with the rs1569723 A/C (|D'| = 0.867) and rs4810485 G/T (|D'| = 0.841). The rs1569723 A/C and rs4810485 G/T were in strong linkage disequilibrium (|D'| = 0.922). By haplotype analyses, we found T-C-T haplotype was associated with a significantly increased risk of SLE as compared with the control group (OR = 1.408; 95 % CI, 1.074–1.845; *P* = 0.013).

**Table 3:** Haplotype distribution in the patients with SLE and controls

CD40 gene (rs1883832/rs1569723/rs4810485) haplotypes	SLE patients 2 n = 410 (%)	Controls 2 n = 440 (%)	OR (95 % CI)	*P* value
T-C-T	211 (51.5)	189 (43.0)	1.408 (1.074–1.845)	0.013
C-A-G	176 (42.9)	207 (47.0)	0.847 (0.646–1.110)	0.228
C-A-T	8 (2.0)	13 (3.0)	0.735 (0.253–2.138)	0.346
C-C-G	6 (1.5)	14 (3.2)	0.452 (0.172–1.187)	0.099
C-C-T	4 (1.0)	8 (1.8)	0.532 (0.159–1.780)	0.298
T-A-T	5 (1.2)	9 (2.0)	0.591 (0.196–1.779)	0.344

### Association between CD40 gene polymorphisms and sCD40 levels

**Fig. 2:**
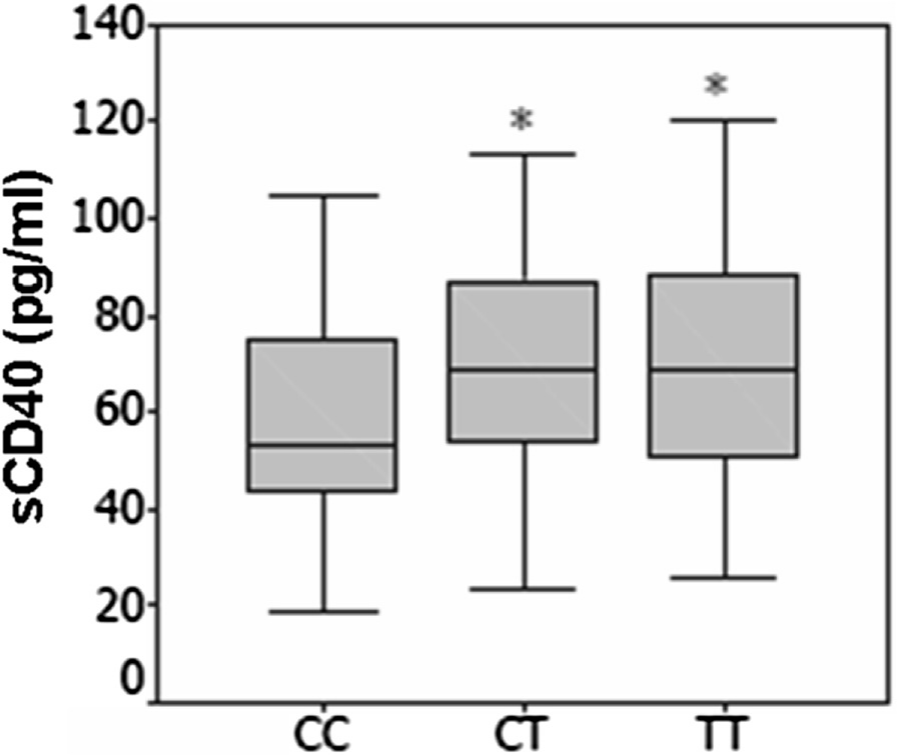
Association between the levels of CD40 and the rs1883832 C/T polymorphism of CD40 gene was observed in patients with SLE. Plasma CD40 levels with CC homozygous were significantly lower than that of the TT homozygous or CT heterozygotes, respectively. However, there were no significant differences in the plasma CD40 levels between CT and TT genotypes

Genotype at the rs1883832 C/T polymorphism was significantly associated with sCD40 levels in patients with SLE. The plasma CD40 levels were significantly higher in individuals with homozygous TT genotypes (62.6 +/− 23.3 pg/ml, *n* = 53) or heterozygous of CT genotypes (59.9 +/− 22.6 pg/ml, *n* = 105) than homozygous of CC genotypes (50.7 +/− 20.4 pg/ml, *n* = 47, *P* < 0.01, respectively). However, there were no significant differences in the plasma CD40 levels between TT and CT genotypes (Fig. 2). In addition, there were no significant associations of the CD40 rs1569723 A/C and rs4810485 G/T polymorphisms with plasma levels of CD40 (data not shown).

## Discussion

CD40, the receptor for CD40L, is a 48-kDa transmembrane protein belonging to the TNF (tumor necrosis factor) superfamily, and is expressed on B cells, endothelial cells, macrophages, dendritic cells, T cells, and fibroblasts. Until now, little information has addressed the association between CD40 polymorphisms and its soluble level in Chinese patients. In this study, we focused on identifying a genetic marker that may help refine the SLE risk profile. We found that the rs1883832 C/T polymorphism of CD40 and the levels of sCD40 were significantly associated with the presence of SLE. The rs1883832 C/T polymorphism may affect the levels of sCD40. Moreover, we also found that the rs1883832 C/T polymorphism was in strong linkage disequilibrium with the rs1569723 A/C (|D'| = 0.867) and rs4810485 G/T (|D'| = 0.841). The rs1569723 A/C and rs4810485 G/T were in strong linkage disequilibrium (|D'| = 0.922). Major two haplotype frequencies of the TCT and CAG among the SLE in the present study were 0.515 and 0.429 respectively. By haplotype analyses, we found that TCT haplotype was associated with a significantly increased risk of SLE as compared with the control groups (OR = 1.408; 95 % CI, 1.074–1.845; *P* = 0.013). Our results suggest that the CD40 gene plays a central role in the mechanism of the SLE pathophysiology. Thus, CD40 gene rs1883832 C/T polymorphism may serve as novel genetic markers of susceptibility to SLE in the Chinese population.

SLE is a chronic inflammatory disease of collagen in the skin, of joints, and of internal organs, and is a complex disorder in which multiple genetic variants, together with environmental and hormonal factors, contribute to disease risk. The etiology of SLE remains unknown, and the pathological mechanisms underlying the related organ and tissue damage have not been fully elucidated [26]. Recently, increasing evidence showed that CD40 contributes to the pathogenesis of chronic inflammatory and autoimmune diseases due to its biological activity [27]. In several reports of SLE, CD40 has either been indirectly or directly shown to be a contributing factor to the disease. In one report, Zhang et al. presented that TT genotype carriers showed higher CD40 expression and serum soluble CD40 ncentration in male IS patients [28]. CD40 polymorphisms are also associated with SLE clinical manifestation, mainly nephritis and arthritis [29, 30]. However, Plasma levels of CD40 were significantly elevated in SLE patients in comparison with healthy controls. In the present study, our data also showed that the plasma sCD40 levels were significantly high in SLE patients compared to controls. The results of our study indirectly suggest that CD40 may play a role in patients with SLE. These observations make CD40 an interesting candidate gene for a role in human SLE.

Several studies have investigated associations between genetic variation in the CD40 gene and SLE, but results of these studies have been inconsistent. Vazgiourakis found that CD40 has been identified as a new susceptibility locus in Greek and Turkish patients with SLE. The rs4810485 minor allele T is under-represented in SLE and correlates with reduced CD40 expression in peripheral blood monocytes and B cells, with potential implications for the regulation of aberrant immune responses in the disease, the CD40 gene rs4810485 G/T polymorphisms between the group of patients with SLE and the control group in European-American population (*P* < 0.05) [31]. Meanwhile, Piotrowski reported that there was no apparent relationship in the genotype frequencies of CD40 gene rs4810485 G/T polymorphisms with the risk of SLE in Polish patients as compared to controls (*P* > 0.05) [32]. Our results showed that there were significant differences in the genotype and allele frequencies of CD40 gene rs1883832C/T polymorphism between the group of patients with SLE and the control group (*P* < 0.05). sCD40 levels were increased in patients with SLE compared with controls (*P* < 0.01). The rs1883832C/T polymorphism of CD40 and its sCD40 levels were associated with SLE in the Chinese population. However their findings suggest that the significant variation in prevalence of risk genetic locis among different populations may also explain some of the sizable geographic variation in disease prevalence. The reason for these discrepancies remains unclear, but several possibilities should be considered. First, it may be due to the genetic trait differences; CD40 gene polymorphisms were distinct in specific population, various ethnicities and geographic region. Furthermore, SLE is a multi-factorial disease and individual exposure to various environmental factors, and genetic susceptibility might have caused different results. In addition, the inadequate study design such as non-random sampling and a limited sample size should also be considered. The possible selection bias that might have been present in the hospital-based, case–control study is a relevant issue. Finally, we cannot exclude that the observed association depends on a gene in linkage disequilibrium with the CD40 gene or on the effect of CD40 on another peptide.

So far, investigations on the CD40 gene rs1883832 C/T polymorphism and its soluble level, which are associated with SLE, have not been performed. Our data demonstrated that CD40 gene rs1883832 C/T polymorphism was associated with SLE (*P* < 0.05). Also, the level of sCD40 was found to be elevated in SLE patients (*P* < 0.01). Moreover, genotypes carrying the CD40 rs1883832 C/T variant allele (TT or CT genotype) were associated with increased CD40 levels compared to the homozygous wild-type genotype (CC genotype) in patients with SLE (*P* <0.01). Additionally, our results showed that sCD40 levels were not associated with the polymorphisms of the CD40 in healthy controls. A plausible explanation is that the sCD40 expression is inducible and its expression is upregulated after stimulation and such inflammatory stimulation in healthy controls should be missing. However, we found that individuals carrying the rs1883832 T allele of the CD40 gene rs1883832 C/T polymorphism, which has been associated with increased sCD40 production, were at a significantly increased risk of SLE. This finding suggests an association between CD40 genotypes and its soluble form. We speculate that CD40 gene rs1883832 C/T polymorphismmay exert an impact on its protein metabolism and stability.

## Conclusion

On the basis of these findings, we conclude that the rs1883832 C/T polymorphism of CD40 and the levels of sCD40 were significantly associated with the risk of SLE in the Chinese population. These results suggest that further studies with larger cohorts of patients should be performed to illustrate the correlation of the CD40 gene polymorphism with SLE susceptibility, independently or in combination with other CD40 SNPs and other genes. Because genetic polymorphisms were often vary different between ethnic groups, further studies are also needed to clarify the association of the CD40 polymorphism with the risk of SLE in diverse ethnic populations.

## Abbreviations

SLE: Systemic lupus erythematosus
CI: Confidence interval
OR: Odds ratio
SNPs: Single nucleotide polymorphisms
ELISA: Enzyme linked immunosorbent assay
